# Optimization of Acid Protease Production by *Aspergillus niger* I1 on Shrimp Peptone Using Statistical Experimental Design

**DOI:** 10.1100/2012/564932

**Published:** 2012-04-19

**Authors:** Rayda Siala, Fakher Frikha, Samiha Mhamdi, Moncef Nasri, Alya Sellami Kamoun

**Affiliations:** ^1^Laboratoire de Génie Enzymatique et de Microbiologie, Ecole Nationale d'Ingénieurs de Sfax, Université de Sfax, BP 1173-3038, Sfax, Tunisia; ^2^Faculté des Sciences de Sfax, Université de Sfax, BP 1171-3000, Sfax, Tunisia; ^3^Laboratoire de Génie Enzymatique des Lipases, Ecole Nationale d'Ingénieurs de Sfax, Université de Sfax, BP 1173-3038, Sfax, Tunisia

## Abstract

Medium composition and culture conditions for the acid protease production by *Aspergillus niger* I1 were optimized by response surface methodology (RSM). A significant influence of temperature, KH_2_PO_4_, and initial pH on the protease production was evaluated by Plackett-Burman design (PBD). These factors were further optimized using Box-Behnken design and RSM. Under the proposed optimized conditions, the experimental protease production (183.13 U mL^−1^) closely matched the yield predicted by the statistical model (172.57 U mL^−1^) with *R* ^2^ = 0.914. Compared with the initial M1 medium on which protease production was 43.13 U mL^−1^, a successful and significant improvement by 4.25 folds was achieved in the optimized medium containing (g/L): hulled grain of wheat (HGW) 5.0; KH_2_PO_4_ 1.0; NaCl 0.3; MgSO_4_(7H_2_O) 0.5; CaCl_2_ (7H_2_O) 0.4; ZnSO_4_ 0.1; Na_2_HPO_4_ 1.6; shrimp peptone (SP) 1.0. The pH was adjusted at 5 and the temperature at 30°C. More interestingly, the optimization was accomplished using two cheap and local fermentation substrates, HGW and SP, which may result in a significant reduction in the cost of medium constituents.

## 1. Introduction

Microorganisms are the most important sources for enzymes production. Among these enzymes, proteases account for nearly 60% of the total industrial enzyme market [1, 2]. A large proportion of commercially available proteases are currently derived from *Bacillus* strains. Nevertheless, the potential use of fungal proteases is being increasingly realized [3]. In fact, fungal enzymes are commonly used in industries due to various technical reasons, including the feasibility of obtaining enzymes at high concentration in the fermentation medium [4]. Fungal proteases offer a distinct advantage over bacterial enzymes in terms of ease of downstream processing [5].

The use of acid proteases has been increased remarkably in various industrial processes such as animal feed, cheese and food processing, and X-ray films [6, 7].

It is well known that extracellular protease production by microorganisms is greatly influenced by media components, especially carbon and nitrogen sources, and physical factors such as temperature, pH, incubation time, agitation, and inoculum density [8–11]. Medium composition is one of the most important parameters when enzymes are produced for industrial purposes particularly, because around 30–40% of the production cost was estimated to be accounted for the cost of the growth medium [12]. Then, the use of local and low cost substrates in growth media can significantly reduce the cost of enzyme production [13]. Fish by-products have been used to a minor extent as a fermentation substrate for protease production, despite their availability in large quantities and their low cost. Ellouz et al. [14] have shown that protease synthesis was strongly induced when *B. subtilis* was grown in media containing only sardinelle heads and viscera powder. Haddar et al. [15] had reported the use of hulled grain of wheat and *Sardinella aurita* peptone for the proteases production by *B. mojavensis* A21. Also, Hadj-Ali et al. [16] demonstrated efficient utilization of both fish powders and hulled grain of wheat as bacterial growth substrates for alkaline proteases production by *B. licheniformis* NH1.

The protease production by *B. cereus* BG1 was stimulated by the use of *S. aurita* powders [17].

Optimization of media compounds by the traditional “one-variable-at-a-time” strategy involving changing one independent variable is the most frequently used operation in biotechnology [18]. This strategy is extremely time-consuming and expensive when a large number of variables are considered and incapable of detecting the true optimum, due especially to the interactions among the factors.

In recent years, the use of statistical approach involving Plackett-Burman designing and Box-Behnken design has gained lot of impetus for medium optimization and for understanding the interactions among various physicochemical parameters using a minimum number of experiments. The Plackett-Burman design allows the screening of main factors, from a large number of variables that can be retained in further optimization process. Box-Behnken design is a collection of statistical techniques for designing experiments, building models, evaluating the effects of factors, and searching optimum conditions of studied factors for desirable responses [19]. Box-Behnken design has been successfully applied in many areas of biotechnology, such as manganese peroxidase production [20], protease production [15, 21], and neomycin production [22].

*Aspergillus niger* I1 has been recently isolated and identified as a producer of an extracellular bleaching stable acid protease [23]. The enzyme has a molecular weight of 49 kDa, it was identified as an aspartic protease, with optimum at pH 3 and 60°C. These properties would make this enzyme potentially useful for industrial applications. In the present study, an effort was done to maximize the acid protease production by *A. niger* I1 by using low-cost fermentation medium. The optimization steps were performed as follows: selecting carbon and nitrogen sources by one-variable-at-a-time approach, screening the main factors influencing protease production using Plackett-Burman design, and assessing the optimal region of the significant variables using Box-Behnken design. 

## 2. Material and Methods

### 2.1. Material

All chemicals used were of analytical grade. Hulled grain of wheat (HGW) was purchased from a local industry. Shrimp flower (SF), shrimp peptone (SP), combined heads and viscera sardinelle (*Sardinella aurita*) powder (CHVSP), and *Mirabilis jalapa* tubers powder (MJTP) were prepared in our laboratory. 

To obtain CHVSP, sardinelle heads and viscera were cooked until boiling, pressed to remove water and fat, minced, and then dried according to Ellouz et al. [14]. MJTP was prepared as described by Hajji et al. [24].

SF was prepared as follows: shrimp wastes, collected from a local fish processing industry, were washed thoroughly with tap water and then cooked for 20 min at 100°C. The solid material obtained was dried, minced to obtain a fine powder, and then stored in a glass bottle at room temperature. The chemical composition (proteins, chitin, lipids, and ash) was determined by Manni et al. [25]. SP was obtained by hydrolysing SF by commercial trypsin.

### 2.2. Microorganism

*A. niger* I1 producing an acid protease was used in the present study. It was identified on the basis of a 650 bp PCR amplified DNA fragment of the 18S rDNA sequence [23]. 

The strain was propagated on potato-dextrose-agar plates at 30°C, and inocula were prepared from 7-days-old mycelia by flooding with 10 mL of sterile distilled water and scraping off the agar plates.

### 2.3. Carbon and Nitrogen Sources Selection

Initial screening of the most significant carbon and nitrogen sources allowing the maximum protease production was performed by one-variable-at-a-time approach. Seven different nitrogen sources (5 g/L), including casein peptone, meat peptone, urea, gelatine, sodium chloride, yeast extract, SP or ammonium sulphate and eight simple or complex carbon sources (10 g/L) including glucose, maltose, lactose, HGW, MJTP, SF, CHVSP, and casein, were tested. Initial M1 medium consists of (g/L): HGW 10.0; (NH_4_)_2_SO_4_ 5.0; CaCl_2_(7H_2_O) 0.4; KH_2_PO_4_ 1.0; Na_2_HPO_4_ 0.8; MgSO_4_(7H_2_O) 0.5; ZnSO_4_ 0.1; NaCl 0.3. Media were autoclaved at 120°C for 20 min. Cultures were inoculated with 10^7^ spores/mL in 300 mL Erlenmeyer flasks with a working volume of 50 mL and incubated in a rotatory shaker (200 rpm) for 72 h. Cultures were centrifuged at 8,000 ×g for 15 min to remove fungi mycelia, and the supernatant was used for estimation of proteolytic activities. It will be designated in the text by enzyme preparation.

All experiments were carried out in duplicate and repeated at least twice.

### 2.4. Estimation of the Fungal Growth

Growth was estimated by the determination of the mycelium dry weight. The fungal mycelia were harvested by centrifugation at 8,000 ×g for 15 min. The pellet was washed with autoclaved bidistilled water, and the dry weight was determined after heating at 105°C until a constant weight [26].

### 2.5. Protease Assay

Protease activity was measured by the method of Kembhavi et al. [27] using hemoglobin as a substrate. Enzyme preparation (0.5 mL), suitably diluted, was mixed with 0.5 mL of hemoglobin 1% (w/v) in 100 mM glycine-HCl (pH 3.0) and then incubated for 5 min at 60°C. The reaction was stopped by adding 0.5 mL trichloroacetic acid 8% (w/v). The mixture was allowed to stand at room temperature for 15 min and then centrifuged at 10,000 ×g for 15 min to remove the precipitate. The absorbance of the soluble fraction was estimated at 280 nm. A standard curve was generated using tyrosine solutions at 0–50 mg/L. One unit of protease activity was defined as the amount of enzyme required to liberate 1 *μ*g of tyrosine per min under the experimental conditions.

### 2.6. Plackett-Burman Design

The Plackett-Burman design is an efficient way to screen the main physicochemical parameters, required for elevated production, among a large number of process variables [28]. The carbon and nitrogen sources, which had been screened earlier, were added to the main culture medium for optimization. The Plackett-Burman method allows evaluation of *N* variables in *N* + 1 experiments; each variable was examined at two levels: (−1) for a low level and (+1) for a high level.  Table 1 illustrates the factors under investigation as well as levels of each factor used in the experimental design. Whereas Table 2 represents the design matrix “Design Expert 7.0” Stat-Ease, Inc., Minneapolis, USA, and was used to analyze the experimental Plackett-Burman design.

### 2.7. Experimental Design

A Box-Behnken design of RSM was employed to optimize the three most significant factors (KH_2_PO_4_, pH, and temperature) for enhancing protease production, screened by Plackett-Burman design, the three independent factors were investigated at three different levels (−1, 0, +1), and the experimental design used for study is shown in Table 3. The protease production was fitted using a second-order polynomial equation, and multiple regression of the data was carried out for obtaining an empirical model related to the most significant factors. The general form of the second-order polynomial equation is

(1) $$\;Y=\beta_{0}+\sum \beta_{i}x_{i}+\sum \beta_{ii}x_{i}^{{}_{2}}+\sum \beta_{ij}x_{i}x_{j},$$

where *Y* is the predicted response, *x*_*i*_ and *x*_*j*_ are independent factors, *β*_0_ is the intercept, *β*_*i*_ is the linear coefficient, *β*_*ii*_ is the quadratic coefficient, and *β*_*ij*_ is the interaction coefficient. 

Design-Expert, version 7.0 (STAT-EASEinc, Minneapolis, USA), was used for experimental designs and statistical analysis of the experimental data. The analysis of variance (ANOVA) was used to estimate the statistical parameters.

### 2.8. Biochemical Properties of the Crude Enzyme Preparation

#### 2.8.1. Effect of pH and Temperature on Enzyme Activity and Stability

The optimum pH of the enzyme preparation was studied over a pH range of 3.0–9.0 at 50°C using hemoglobin 1% (w/v). For studying pH stability, the crude enzyme was incubated in buffers of different pH values in the range of pH 3.0–9.0 for 1 h at 30°C. Residual proteolytic activity was then determined under standard assay conditions. The following buffer systems were used: 100 mM glycine-HCl buffer for pH 3.0, 100 mM sodium acetate buffer for pH 4.0–6.0, 100 mM potassium phosphate buffer for pH 7.0, 100 mM Tris-HCl buffer for pH 8.0, and 100 mM glycine-NaOH buffer for pH 9.0.

To investigate the effect of temperature, proteolytic activity was tested at different temperatures using hemoglobin as a substrate for 5 min at pH 4.0. Thermal stability was examined by incubating the enzyme for 60 min at 30, 40, 50, and 60°C. Aliquots were withdrawn at desired time intervals to test the remaining activity at optimum conditions of pH and temperature. The nonheated enzyme was considered as the 100% control.

#### 2.8.2. Effects of Metal Ions and Inhibitors on Proteolytic Activity

The effect of various metal ions at 5 mM on enzyme activity was investigated using CaCl_2_, MnSO_4_, ZnSO_4_, CoSO_4_, CuSO_4_, BaCl_2_, FeCl_2_, HgCl_2_, MgSO_4_, NaCl, and KCl.

The effects of inhibitors on protease activity were studied using phenylmethylsulfonyl fluoride (PMSF), pepstatin A, *β*-mercaptoethanol, and ethylenediaminetetraacetic acid (EDTA). The enzyme preparation was preincubated with inhibitor for 60 min at 4°C, and then the remaining proteolytic activity was estimated using hemoglobin as a substrate. The activity of the enzyme without any additive was taken as the 100% control.

#### 2.8.3. Zymography

For zymography, casein was used as substrate. Casein-zymography was performed to estimate the number of the proteolytic activity in the crude supernatant. Zymography was performed in conjunction with SDS-PAGE according to the method of Garcia-Carreno et al. [29] with a slight modification. SDS-PAGE was performed as described by Laemmli [30], using a 5% (w/v) stacking gel and a 12% (w/v) separating gel. The samples were not heated. After electrophoresis, the gel was submerged in 100 mM glycine-HCl pH 3.0 (buffer A) containing 2.5% Triton X-100 for 60 min, with constant agitation to remove SDS. Triton X-100 was then removed by washing the gel three times with buffer A. The gel was then incubated with 1% (w/v) casein in buffer A for 15 min at 50°C. Finally, the gel was stained with Coomassie brilliant blue R-250 for zymography analysis. The development of clear zone on the blue background of the gel indicated the presence of protease activity. The molecular mass markers used in SDS-PAGE were phosphorylase b (97 kDa); albumin (66 kDa); ovalbumin (45 kDa); carbonic anhydrase (30 kDa); trypsin inhibitor (20.1 kDa); bovine *α*-lactoalbumin (14.4 kDa).

## 3. Results

### 3.1. Selection of Carbon and Nitrogen Sources

**Figure 1:**
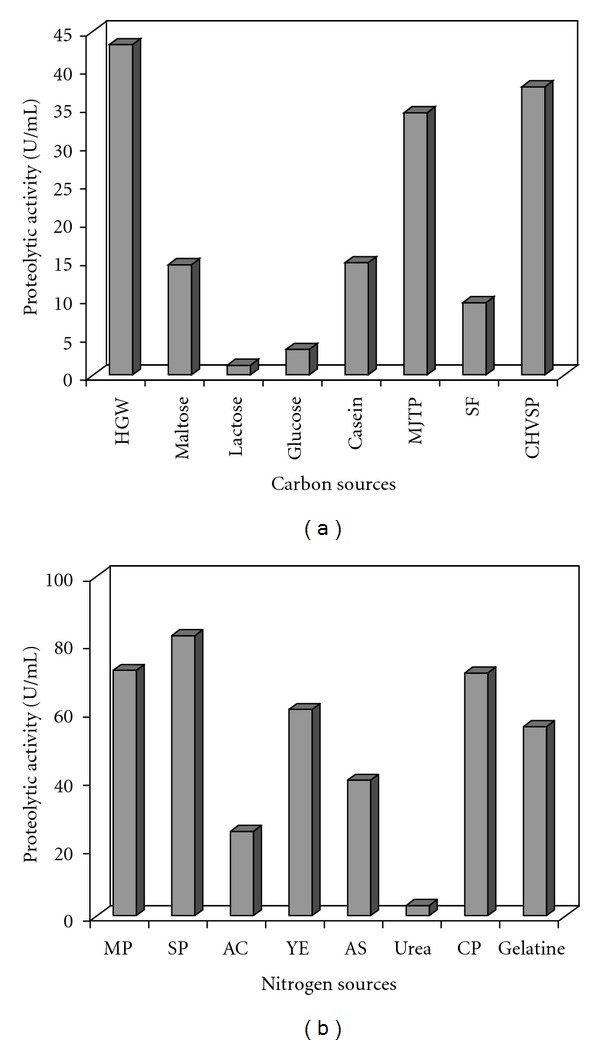
Effects of carbon sources at 10 g/L (a); nitrogen sources at 5 g/L (b) on the production of proteolytic activity by *A. niger* I1. HGW: hulled grain of wheat, MJTP: *Mirabilis jalapa* tuber powder, SF: shrimp flower, CHVSP: combined heads and viscera sardinelle powder, MP: meat peptone, SP: shrimp peptone, AC: ammonium chloride, YE: yeast extract, AS: ammonium sulphate, CP: casein peptone.

A series of experiments was first carried out to study the effects of various simple and complex carbon and nitrogen sources on protease production by *A. niger*. Cultures were first conducted in medium M1 containing different carbon sources, each added at a concentration of 10 g/L. Protease activity was produced at high levels in the presence of complex organic carbon sources (Figure 1(a)). The highest level was obtained with HGW (43.13 U mL^−1^) followed by CHVSP and MJTP (37.34 and 34.24 U mL^−1^, resp.). HGW and MJTP were tested as carbon sources since they contained high starch content 62 and 32.6 ± 2%, respectively. The easily assimilated simple carbon sources like maltose, glucose, and lactose resulted in weak acid protease production, 14.37, 3.33, and 1.24 U mL^−1^, respectively.

 In general, both organic and inorganic nitrogen sources were used efficiently for protease production. In the present study, six organic (yeast extract, casein peptone, gelatine, CHVSP, meat peptone, and SP) and two inorganic nitrogen sources (ammonium sulphate and ammonium chloride) were tested, at a concentration of 5 g/L, in M1 medium containing HGW at 10 g/L as carbon source (Figure 1(b)). Among the various nitrogen sources studied, *A. niger* I1 produced high levels of protease on SP (82.3 U mL^−1^) followed by meat peptone, casein peptone, CHVSP, yeast extract, and gelatine (72.67, 71.63, 68.75, 61.24, and 55.68 U mL^−1^, resp.). Ammonium sulphate and ammonium chloride showed weak acid protease production levels, 39.8 and 25.49 U mL^−1^, respectively.

Among the various carbon and nitrogen sources tested, HGW and SP were found to be the most suitable substrates for the production of acid protease by *A. niger* I1. Thus, these substrates were selected for further optimization steps.

### 3.2. Selection of Significant Variables by Plackett-Burman Design

Analysed by design Expert, a first-order model was fitted to results obtained from the twelve experiments.

The *t*-test was used to identify the effect of each factor on protease production.  Table 4 shows that KH_2_PO_4_, initial pH, and temperature are the most significant factors (*P* < 0.05); they were selected for further optimization.

According to Table 4, temperature, KH_2_PO_4_, and initial pH were determined to be significant factors (*P* < 0.05) on the protease production. Among nonsignificant factors, six factors (HGW, NaCl, MgSO_4_, CaCl_2_, ZnSO_4_, and inoculum's size IS) exerted a negative effect while two variables (Na_2_HPO_4_ and SP) exerted positive effects on proteases production. The significant variables (temperature, initial pH, and KH_2_PO_4_) were selected for further optimization by a response surface methodology. The nonsignificant variables with positive effect (Na_2_HPO_4_ and SP) were fixed at high levels, while variables with a negative effect (HGW, NaCl, MgSO_4_, CaCl_2_, ZnSO_4_, and IS) were fixed at low levels.

### 3.3. Optimization of Significant Variables Using RSM

Box-Behnken design was used to determine the optimum values of the three selected significant variables (temperature, initial pH, and KH_2_PO_4_ concentration) for the protease production. A total of 17 experiments with different combinations of the three selected variables were performed. The design matrix with the corresponding results of Box-Behnken experiments, as well as the predicted results, is presented in Table 4. The *P* values for the model (<0.0001) and for “Lack of Fit” (0.0687) also suggested that the obtained experimental data was a good fit with the model. The regression equation coefficients were calculated, and the data were fitted to a first-order polynomial equation. The response of protease production (*Y*) by *A. niger* I1 can be expressed in terms of the following regression equation:

(2) $$Y=+55.20-3.83X_{1}+12.20X_{3}-8.16X_{1}X_{3},$$

where *X*_1_ is KH_2_PO_4_ concentration; *X*_3_ is temperature.

The ANOVA analysis of the optimization study (Table 5) indicated that, among the three significant variables selected by the Plackett-Burman design experiment, KH_2_PO_4_ and temperature were found to have a significant effect on enzyme production contrary to pH. Furthermore, the interaction between KH_2_PO_4_ and temperature (*X_1_X_3_*) was significant, as was shown by the low *P* value (0.0001) for the interactive terms.

The regression equation obtained from the ANOVA analysis showed that the value of *R* ^2^ (multiple correlation coefficient) is 0.914. This value indicated that only 10.884% of the total variation was not explained by the model. The value of the adjusted determination coefficient (Adj-*R* ^2^ = 0.894) further confirms the significance of the model. The value of the predicted determination coefficient pred*-R^2^* of 0.7841 is in reasonable agreement with the value of the adjusted determination coefficient Adj*-R^2^* of 0.8939. In addition, the model has an adequate precision value of 24.832. This suggests that the model can be used to navigate the design space (Table 6).

The lower reliability of the experiment is usually indicated by the high value of the coefficient of variation (CV) [31]. In the present case, a low CV (6.126%) denotes that the experiments performed are highly reliable as was shown by Mohanasundararaju et al. [32].

**Figure 2:**
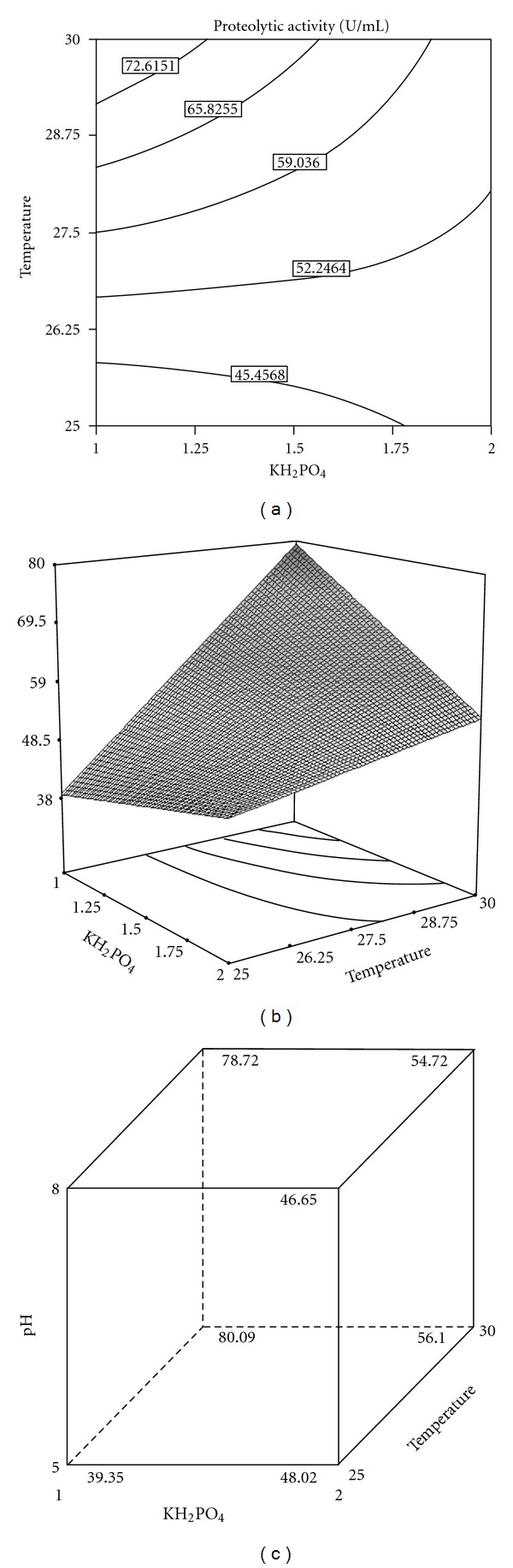
Response surface plot of proteolytic enzyme production showing the interactive effects of the temperature and KH_2_PO_4_ concentrations (a, b), and initial pH, temperature, and KH_2_PO_4_ concentrations (c).

Response surface plot was generally the graphical representation of the regression equation, from which the response (protease production) is plotted against any two variables (Figures 2(a), 2(b), and 2(c)), while other variables were fixed at their middle levels.

As shown in Figures 2(b) and 2(c), protease production increased only when temperature increased and could not increase with KH_2_PO_4_ concentration. The model predicted that the maximum proteolytic enzyme production of 172.57 U mL^−1^ is achieved in the medium containing (g/L): HGW 5; SP 5; KH_2_PO_4_ 1; Na_2_HPO_4_ 1.6; CaCl_2_(7H_2_O) 0.4; ZnSO_4_ 0.1; NaCl 0.3; MgSO_4_(7H_2_O) 0.5; pH 5.0.

### 3.4. Model Validation

The experimentally determined production values were found to be in good agreement with the statistically predicted ones (*R* ^2^ = 0.914), confirming the model's authenticity. In addition, the “Pre-*R* ^2^” of 0.7841 is in reasonable agreement with the “Adj-*R* ^2^” of 0.8939, indicating that this model can be used to navigate the design space (Table 6).

### 3.5. Casein Zymography

**Figure 3:**
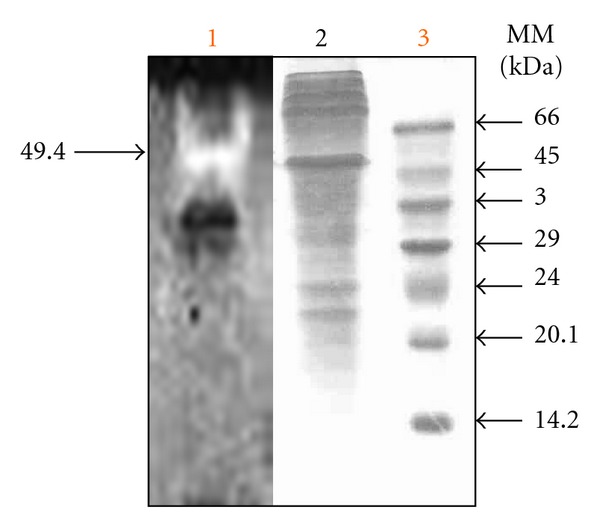
SDS-PAGE and zymography of *A. niger* I1 crude enzyme preparation. Lane 1: zymography on casein; lane 2: SDS-PAGE after staining with Coomassie blue R250; lane 3: molecular mass markers.

Casein zymography of the crude enzyme preparation revealed the presence of one clear zone, suggesting the presence of one acid protease (Figure 3). The molecular mass of the *A. niger* I1 protease was estimated to be 49.4 kDa by SDS-PAGE.

### 3.6. Biochemical Properties of Proteolytic Enzyme

#### 3.6.1. Effect of pH on Enzyme Activity and Stability

**Figure 4:**
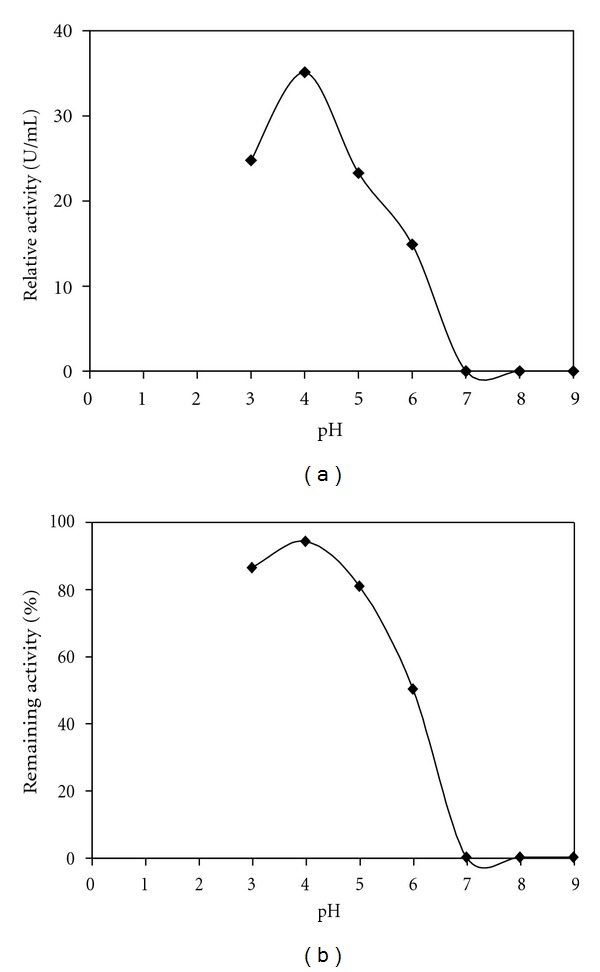
Effect of pH on the activity (a) and stability (b) of the extracellular proteases of *A. niger* I1 strain. pH optima were determined by incubating the crude enzyme with the substrate at different pH values at 60°C. The maximum activity obtained at pH 3.0 with hemoglobin as substrates was considered to be 100%. The pH stability was determined by incubating the crude enzyme in different buffers for 1 h at 4°C, and the residual activity was measured at pH 3.0 and 60°C with hemoglobin as a substrate. The activity of the enzyme before incubation was taken as 100%.

The effect of pH on the activity of the crude enzyme was determined over a pH range of 3.0–9.0 at 60°C using hemoglobin as substrate. As shown in Figure 4(a), the *A. niger* I1 enzyme preparation exhibited maximum activity at pH 4.0.

The pH stability of the crude enzyme was studied by assaying the residual activity of the proteolytic enzyme after incubation at 4°C for 1 h in buffers of various pH values. The crude enzyme was highly stable over a wide pH range, maintaining more than 86 and 80% of its initial activity at pH 3.0 and 5.0, respectively (Figure 4(b)). However, proteolytic enzyme activity decreased significantly below pH 6. The enzyme was inactivated at pH 7, 8, and 9.

#### 3.6.2. Effect of Temperature on Enzyme Activity and Stability

**Figure 5:**
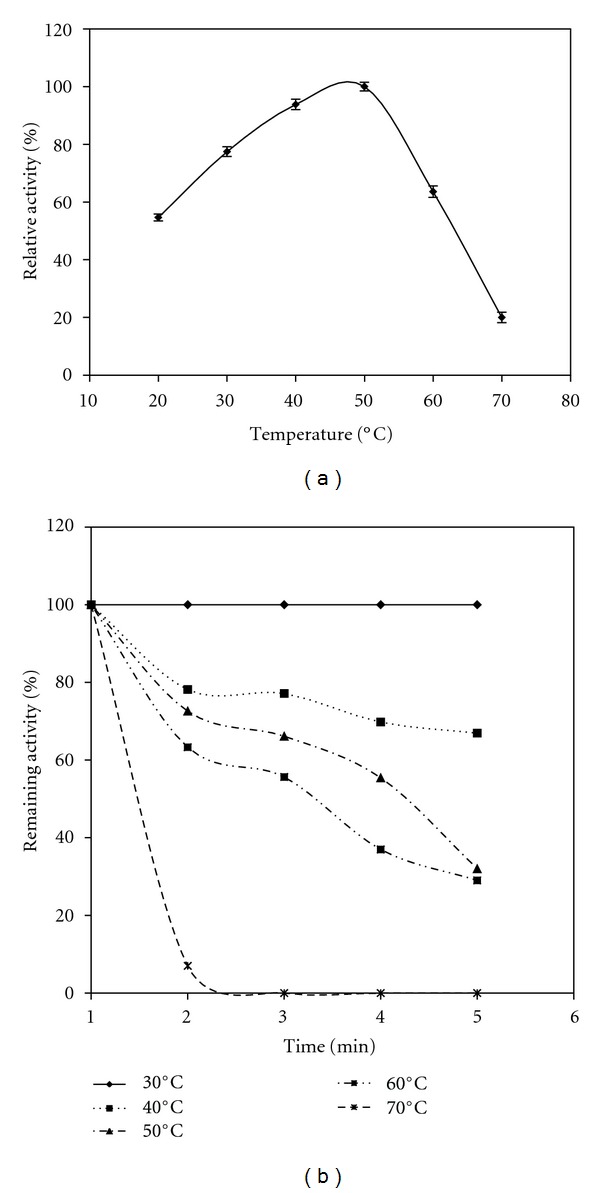
Effect of temperature on the activity (a) and stability (b) of the extracellular proteinases of *A. niger* I1. The temperature profile was determined by assaying proteolytic activity at temperatures between 30°C and 70°C. The activity of the enzyme at 50°C and pH 4.0 using hemoglobin as a substrate was taken as 100%. The temperature stability was determined by incubating the crude enzyme in different temperature for 1 h, and the residual activity was measured at pH 4.0 and 50°C with hemoglobin as a substrate. The nonheated enzyme was considered as 100% control.

The influence of temperature on the crude supernatant was examined at pH 4.0 using hemoglobin as substrate. The temperature activity profile showed that the crude enzyme was highly active between 30°C and 60°C with an optimal activity at 50°C using hemoglobin as substrate (Figure 5(a)).

To examine the thermal stability of *A. niger* I1 protease, the enzyme preparation was incubated at pH 4, at various temperatures for different time periods, and then the residual activities were measured. The thermal stability profile of the crude enzyme showed that the enzyme is highly stable at temperatures below 30°C (Figure 5(b)). At 30°C, the enzyme remains fully active even after 1 h incubation. The enzyme was rapidly inactivated at 70°C, losing 93% of its initial activity after 15 min incubation.

#### 3.6.3. Effects of Metal Ions and Enzyme Inhibitors on Enzyme Activity

The effects of various metal ions (5 mM) on the proteolytic activity of the enzyme preparation were studied at pH 4.0 and at 50°C by the addition of the respective cations or enzyme inhibitor to the reaction mixture. As shown in Table 7, the addition of K^+^ increased slightly proteolytic activity. However, Hg^2+^ and Cu^2+^ inhibited the proteolytic activity by 22% and 39.12%, respectively. Ca^2+^ and Zn^2+^ had no influence on enzyme activity. The crude enzyme was completely inhibited by pepstatin A. PMSF and EDTA are practically without influence on the activity of the crude enzyme.

## 4. Discussion and Conclusion

In general, no defined medium has been carried out for the optimum production of proteases from different microbial strains [33]. Each microorganism has its own special conditions for the maximum enzyme production. Several researchers attempted to induce protease production by using glucose or starch, coupled with expensive nitrogen sources such as yeast extract, peptone, or casamino acids. However, few studies have been made to induce protease production using inexpensive carbon and nitrogen sources [34–36]. Since shrimp wastes are free, abundant, and polluting the environment, their utilization as substrate by *A. niger* could result in a twofold benefit, namely, a substantial reduction in the cost of enzyme production and a hygienic treatment of the environment. Indeed, in the present work, shrimp peptone was found to be an excellent substrate for protease induction by *A. niger* I1. This may be due to the fact that shrimp wastes contain carbohydrates and minerals and a great amount (~40%) of proteins [25] and then the resulting peptone provides many useful peptides or other molecules, for protease induction or synthesis.

In general, both organic and inorganic nitrogen sources were used efficiently for protease production. The optimum proteolytic activity produced by *A. niger* I1 was achieved with shrimp peptone as nitrogen source (82.3 U mL^−1^), followed by casein peptone (71.63 U mL^−1^) compared to the control with ammonium sulphate as nitrogen source (39.80 U mL^−1^). The addition of ammonium chloride or urea to the medium containing HGW as carbon source decreased protease synthesis to 25.49 and 3.66 U mL^−1^, respectively. Through these results, it can be concluded that shrimp peptone is an excellent source of nitrogen for production of extracellular proteases by *A. niger* I1.

Hajji et al. [24] studies have established that extracellular protease secretion by *A. clavatus* ES1 is substantially influenced not only by carbon and nitrogen sources, but also by initial pH and temperature of the growth.

Indeed, in line with these studies, our study showed that KH_2_PO_4_ concentration, temperature, and initial pH were found to influence enzymes synthesis. The optimization of these three parameters (KH_2_PO_4_ 1.0 g/L, pH 5, and 30°C) resulted in a proteolytic activity of 183.13 U mL^−1^, with a 2.2 increase comparing to 82.3 U mL^−1^ obtained on the unoptimized medium containing 10.0 g/L HGW and 5 g/L SP.

Our findings are in accordance with those of Luciana and Suto [37] who demonstrated that KH_2_PO_4_ concentration was the most influential variable on proteolytic enzyme production by *Cellulosimicrobium cellulans*.

Finally, the maximum proteolytic enzyme production was achieved at the following conditions: temperature 30°C, agitation speed of 150 rpm, pH 5.0, and (g/L) HGW, 5, SP, 5; KH_2_PO_4_, 1, Na_2_HPO_4_, 1.6, CaCl_2_, 0.4, ZnCl_2_, 0.1, NaCl, 0.3, MgSO_4_(7 H_2_O), 0.5; other salts were taken at their low levels as shown in Plackett-Burman design. By optimizing the medium composition and the culture conditions, not only the production of proteases was enhanced by 4.25-fold from 43.13 to 183.13 U mL^−1^ but also the cost of enzyme production was reduced since two cheap and readily available complex fermentation substrates HGW and SP were used.

The use of statistical models to optimize culture medium components and conditions has increased in present-day biotechnology, due to its easy applicability, reliability, and validity. In the present study, the significant variables necessary for the enhancement of proteolytic enzyme production were selected using the Plackett-Burman design. The RSM applied to the optimization of proteases production in this investigation suggested the importance of a variety of factors at different levels. RSM was successfully applied in the production of proteases by *A. awamori* [38] and *A. terreus* [39].

In this study, *A. niger* I1 was found to produce at least only one acid protease as revealed by casein zymography. Van den Hombergh et al. [40] reported at least three aspartic proteases for a strain of *A. niger.* Nevertheless, other works reported the production of only one proteolytic enzyme by *Aspergillus oryzae* MTCC 5341 strain [41].

The enzyme has its optimum activity at pH 4.0, and then protease activity decreased significantly below and above pH 4.0. Optimum pH values between 3.0 and 5.5 have been reported for other fungi proteases, such as the one of *Penicillium camemberti* (pH 3.5) [42] and *Rhizopus oryzae* (pH 5.5) [43]. The *A. niger* NRRL 1785 protease exhibited an optimum at pH 4.0 [44].

The crude enzyme showed maximum activity at 50°C on hemoglobin. The optimum temperature of *A. niger* I1 was similar to those from other fungi proteases, such as the one of *Neosartorya fischeri* var. spinosa IBT 4872 [45]. Proteases from *R. oryzae* [43], *P. duponti* K1014 [46], *P. oxalicum* [47], and *Cryptococcus albidus* [48] exhibited optimum activity at 60°C.

The effects of various metal ions at a concentration of 5 mM on the activity of *A. niger* I1 protease were studied at pH 4.0 and 50°C. *A. niger* I1 acid protease was practically insensitive to the most metallic ions. So this property is in the view of potential food industry applications. None of the metallic ions enhanced protease activity. In this respect, the *A. niger* I1 enzyme resembles to the acid proteases of *Hebeloma crustuliniforme* [49] and *Mucor pusillus* [50]. The effect of a variety of enzyme inhibitors, such as chelating agent and specific group reagents on the activity, was also investigated. Among the cited proteases produced by *A. niger* strains [40, 51, 52], the enzyme can be classified as an acid (aspartic) protease and belonged to the A1 family because of its optimal pH at 4.0 and full inhibition by pepstatin A [53].

In conclusion, this work attempted to demonstrate the efficient use of Plackett-Burman and RSM approaches to determine the conditions leading to enhance extracellular protease production by *A. niger* I1 on two local and low-cost substrates, HGW and SP.

## Figures and Tables

**Table 1:** Levels of the factors tested for the production of proteases by *A. niger* I1 using Plackett-Burman design.

Variables	Unit	Lower level (−1)	Higher level (+1)
Temperature	°C	25	30
Hulled grain of wheat	g/L	5	15
Shrimp peptone	g/L	2	5
KH_2_PO_4_	g/L	1	2
MgSO_4_	g/L	0.5	1
NaCl	g/L	0.3	0.6
pH		5	8
ZnCl_2_	g/L	0.1	0.2
Na_2_HPO_4_	g/L	0.8	1.6
CaCl_2_	g/L	0.4	0.8
Inoculum's size	Sp/mL	5 × 10^6^	10^7^

Sp: spores.

**Table 2:** Plackett-Burman experimental design matrix with proteolytic enzymes production levels.

Variables	*T*	HGW	SP	KH_2_PO_4_	MgSO_4_	NaCl	pH	ZnCl_2_	Na_2_HPO_4_	CaCl_2_	IS	Protease activity
Run	Units											
	°C	g/L	g/L	g/L	g/L	g/L	—	g/L	g/L	g/L	Sp/mL	U/mL

(1)	30	5	5	1	0.5	0.3	8	0.1	1.6	0.4	10^7^	74.11
(2)	30	15	2	2	0.5	0.3	5	0.2	1.6	0.8	5 × 10^6^	60.58
(3)	25	15	5	1	1	0.3	5	0.1	1.6	0.8	10^7^	68.62
(4)	30	5	5	2	0.5	0.6	5	0.1	0.8	0.8	10^7^	78.82
(5)	30	15	2	2	1	0.3	8	0.1	0.8	0.4	10^7^	85.49
(6)	30	15	5	1	1	0.6	5	0.2	0.8	0.4	5 × 10^6^	14.11
(7)	25	15	5	2	0.5	0.6	8	0.1	1.6	0.4	5 × 10^6^	183.13
(8)	25	5	5	2	1	0.3	8	0.2	0.8	0.8	5 × 10^6^	128.43
(9)	25	5	2	2	1	0.6	5	0.2	1.6	0.4	10^7^	74.11
(10)	30	5	2	1	1	0.6	8	0.1	1.6	0.8	5 × 10^6^	52.94
(11)	25	15	2	1	0.5	0.6	8	0.2	0.8	0.8	10^7^	55.29
(12)	25	5	2	1	0.5	0.3	5	0.1	0.8	0.4	5 × 10^6^	96.86

*T*: temperature; HGW: hulled grain of wheat; SP: shrimp peptone; IS: Inoculum's size; Sp: spores.

**Table 3:** The Box-Behnken design of RSM for optimization of protease production.

Variable	KH_2_PO_4_	pH	Temperature	Protease activity
Variable code	*X_1_*	*X_2_*	*X_3_*	
Run	Units			
	(g/L)		(°C)	(U/mL)

(1)	1	5	27.5	58.11
(2)	1	6.5	25	44.11
(3)	1	6.5	30	82.14
(4)	1	8	27.5	57.09
(5)	1.5	8	25	39.64
(6)	1.5	5	25	37.49
(7)	1.5	5	30	67.17
(8)	1.5	8	30	64.19
(9)	2	5	27.5	56.82
(10)	2	8	27.5	53.17
(11)	2	6.5	25	47.72
(12)	2	6.5	30	53.09
(13)	1.5	6.5	27.5	56.5
(14)	1.5	6.5	27.5	57.72
(15)	1.5	6.5	27.5	52.66
(16)	1.5	6.5	27.5	54.47
(17)	1.5	6.5	27.5	56.39

**Table 4:** Identification of significant variables for proteolytic enzymes production by *A. niger* I1 using Plackett-Burman design.

Variables	*t*-ratio	*P *value
Intercept	17.16	<0.0001
Temperature	−3.67	0.0032*
HGW	−1.88	0.0847
SP	0.75	0.4704
KH_2_PO_4_	3.84	0.0024*
MgSO_4_	−1.16	0.2698
NaCl	−1.18	0.2601
Initial pH	2.20	0.0478*
ZnCl_2_	−1.92	0.0787
Na_2_HPO_4_	0.44	0.6682
CaCl_2_	−1.36	0.2000
Inoculum's size	−0.03	0.9760

*Statistically significant at 95% of confidence level.

**Table 5:** Analysis of variance for response surface 2FI model (partial sum of squares, type III).

Source of variation	S.S	D.F	M.S	*F* value	*P*-value	Significant
Model	1587.64	6	264.61	19.3708	<0.0001	*Significant
*X_1_*	117.43	1	117.43	8.5964	0.0150	
*X_2_*	3.78	1	3.78	0.2768	0.6103	
*X_3_*	1191.45	1	1191.45	87.2217	<0.0001	
*X_1_X_2_*	1.73	1	1.73	0.1266	0.7294	
*X_1_X_3_*	266.67	1	266.67	19.5218	0.0013	
*X_2_X_3_*	6.58	1	6.58	0.4816	0.5035	
Residual	136.60	10	13.66			
Lack of fit	120.77	6	20.13	5.0842	0.0687	Not significant
Pure error	15.84	4	3.96			

Total	1724.24	16				

S.S: sum of squares; D.F: degree of freedom; M.S: mean square.

*Statistically significant at 95% of confidence level.

**Table 6:** Variance analysis (ANOVA) of the model 2FI (partial sum of squares, type III).

Standard deviation	3.382
Average deviation	55.205
Variation coefficient %	6.126
Predicted residual sum	372.186
*R* ^2^	0.914
Adj-*R* ^2^	0.894
Pred-*R* ^2^	0.784
Precision	24.832

**Table 7:** Effect of some metal ions and inhibitors on *A. niger* I1 protease activity.

Chemicals	Concentration (mM)	Relative activity (%)
Control	—	100
Ca^2+^	5	100 ± 2
Mg^2+^	5	86
Mn^2+^	5	85 ± 3
Cu^2+^	5	61 ± 4
Ba^2+^	5	87
Hg^2+^	5	78 ± 2
Na^+^	5	93 ± 2
K^+^	5	110 ± 3
Zn^2+^	5	97
PMSF	5	100
EDTA	5	100
DTNB	5	100
Pepstatin A	1.5 × 10^−3^	0
*β*-mercaptoethanol	5	100 ± 3
